# Simulated NIR spectra as sensitive markers of the structure and interactions in nucleobases

**DOI:** 10.1038/s41598-019-53827-6

**Published:** 2019-11-22

**Authors:** Krzysztof B. Beć, Justyna Grabska, Yukihiro Ozaki, Mirosław A. Czarnecki, Christan W. Huck

**Affiliations:** 1 Institute of Analytical Chemistry and Radiochemistry, Leopold-Franzens University, Innrain 80/82, CCB-Center for Chemistry and Biomedicine, 6020 Innsbruck, Austria; 2 Department of Chemistry, School of Science and Technology, Kwansei Gakuin University, Sanda, Hyogo, 669-1337 Japan; 3 Faculty of Chemistry, University of Wrocław, F. Joliot-Curie 14, 50-383 Wrocław, Poland

**Keywords:** Biophysical chemistry, Near-infrared spectroscopy

## Abstract

Near-infrared (near-IR; NIR) spectroscopy is continuously advancing in biophysical and biochemical fields of investigation. For instance, recent progresses in NIR hyperspectral imaging of biological systems may be noted. However, interpretation of NIR bands for biological samples is difficult and creates a considerable barrier in exploring the full potential of NIR spectroscopy in bioscience. For this reason, we carried out a systematic study of NIR spectra of adenine, cytosine, guanine, and thymine in polycrystalline state. Interpretation of NIR spectra of these nucleobases was supported by anharmonic vibrational analysis using Deperturbed Vibrational Second-Order Perturbation Theory (DVPT2). A number of molecular models of nucleobases was applied to study the effect of the inter-molecular interactions on the NIR spectra. The accuracy of simulated NIR spectra appears to depend on the intra-layer interactions; in contrast, the inter-layer interactions are less influential. The best results were achieved by combining the simulated spectra of monomers and dimers. It is of particular note that in-plane deformation bands are far more populated than out-of-plane ones and the importance of ring modes is relatively small. This trend is in contrast to that observed in mid-IR region. As shown, the local, short-range chemical neighborhood of nucleobase molecules influence their NIR spectra more considerably. This suggests that NIR spectra are more sensitive probe of the nucleobase pairing than mid-IR ones. The obtained results allow, for the first time, to construct a frequency correlation table for NIR spectra of purines and pyrimidines.

## Introduction

Nucleobases (nucleic acid bases) are one of the key building blocks of life and hence attract high attention^1–3^. Vibrational spectroscopy is a frequently used tool in biochemical research concerning nucleobases and their derivatives^4,5^. It offers high sensitivity, selectivity, structural specificity, and non-destructive sample probing^6^. One can study molecules not only in the gas-phase, solution, solid-state, or matrices, but also in biological samples^7^. These studies provide valuable information about the intermolecular interactions^8^, base pairing^9^, and tautomerization^10^ of nucleobases. IR and Raman spectroscopy have been used extensively in the investigations of the structure, interactions and properties of nucleobases and related compounds^11–13^. In contrast, investigations of near-infrared (near-IR; NIR) spectra of nucleobases are very rare^14,15^. Though, the experimental NIR spectra of some nucleobases have been known for a long time^16^, any comprehensive exploration of these spectra have not been undertaken as yet. In particular, NIR band assignments of nucleobases have never been carried out in detail, hence an understanding of NIR spectra of nucleobases not only in solutions but also in well-defined crystalline phases is still unsatisfactory. A number of open questions remain on the relation between structural and intermolecular properties of nucleobases and their NIR spectra. This knowledge is essential because the conformational flexibility of nucleobases has an important impact on their functional properties^17,18^.

NIR spectroscopy offers a number of advantages as compared with the other spectral regions^19–21^. This method provides useful information on the molecular structure, interactions, dynamics and anharmonicity^22–25^. However, major focus is directed towards applications of NIR spectroscopy in bioscience^26^, medicine^27,28^, chemical analysis^29^, and spectral imaging^30–32^ NIR bands are weak; typical absorption coefficients of the first overtones are smaller by up to 10^2^ than those corresponding to mid-IR (MIR) fundamental peaks^33^. In the case of higher overtones, this difference may even exceed 10^3^ ^33^. This is a notable advantage of NIR spectroscopy in many applications. The eventuality of a complete absorption of the radiation by a sample is far lesser, unlike in mid-IR spectroscopy that requires sample preparation or attenuated total reflection (ATR) approach^34^. For instance, novel NIR hyperspectral imaging approaches are capable of examining entire organisms^35–37^. Real-time measurements of dynamic processes such as blood flow have recently become possible^38^. NIR spectroscopy is suitable for non-destructive investigations of the samples with high water content as well^35^. These factors contribute to a remarkable potential of the method in biomedical applications^39^. However, compared with mid-IR and Raman, NIR spectroscopy suffers from a poor chemical specificity, which is one of the most important problems to be addressed in current research^40^. This is particularly significant in biological applications, in which complex spectra of biomolecules are analyzed. Therefore, the fundamental research aimed on more comprehensive understanding of NIR spectra is an important step towards increasing the chemical specificity of NIR spectroscopy.

A complexity of NIR spectra is a considerable barrier in several practical applications of NIR spectroscopy^41^. Despite of numerous efforts, classical methods of spectral assignments in NIR region meet considerable limitations^40^. Hence, computational chemistry is a powerful tool offering robust, detailed and independent insight into the origin of NIR bands. A remarkable progress in computational chemistry made possible for comprehensive exploration of NIR spectra^42^. Highly accurate simulations of small- to medium-size molecules in solutions opened the way to exploration of more complex systems^42–44^. Quantum chemical calculations have been applied in studies of NIR spectra of basic molecules like alcohols^45^, phenols^46^, nitriles^47^, or carboxylic acids^48,49^. These studies cover also a series of important molecules including melamine^50^, thymol^51^, rosmarinic acid^52^, short-^53^, medium-^54^ and long-chain fatty acids^55^. These investigations provided interesting information on the effects of isotopic substitution^56,57^, rotational isomerism^23,45,48,57^, liquid structure^47^, conformational equilibria^23^, and hydrogen-bonding^47,49^.

Highly efficient vibrational second-order perturbation theory (VPT2) is particularly useful for computational studies of medium-size or larger molecules^54,55^. Therefore, this method has commonly been applied for anharmonic vibrational analyses of nucleobases and their complexes. Deperturbed and generalized variants of VPT2 method (DVPT2/GVPT2) have been adopted for detailed studies of IR features of numerous molecules^58^, including nucleobases^59–63^. For example, uracil has been examined by the second-order operator canonical Van Vleck perturbation theory (CVPT2)^64^. On the other hand, accurate but computationally highly expensive variational approach has recently been optimized in resource usage by Thomas *et al*.^65^. The authors applied their hierarchical intertwined reduced-rank block power method (HI-RRBPM) to uracil case^65^. However, the previous anharmonic calculations of nucleobases have primarily been focused on IR region, and the results have been compared with the gas-phase spectra^59–65^. Anharmonic calculations are gaining an increasing importance nowadays, with various methods being used for this purpose. Exploration of anharmonicity and crystal packing effects in mid-IR and Raman spectra attracts much attention because of the importance of understanding fine shifts, splitting and intensity redistribution upon the formation of weak intermolecular interactions, as for instance demonstrated by Minaeva *et al*.^66–68^ Prediction of overtones and combination bands helps an interpretation of vibrational optical activity spectra. Recently, combined approaches have been applied in Raman optical activity (ROA) and vibrational circular dichroism (VCD) spectroscopy, and computational investigations significantly extended the potential of these studies by providing insights on intermolecular interactions of chiral molecules and liquids^69,70^. Yet, these recent works focused on mid-IR, while theoretical investigations in NIR region remain rare.

The major purpose of the present study is the theoretical reproduction of NIR spectra of polycrystalline nucleobases (adenine, guanine, cytosine and thymine). In addition, we elucidate the effect of chemical neighborhood on NIR spectra of these nucleobases. The correlation between the intermolecular interactions and hydrogen-bonding of nucleobases and NIR spectra has never been attempted before^8,9^. This problem is interesting in a wider context, e.g. interactions between biomolecules and water^35^. Furthermore, we intend to explore the potential of the present approach to study important biomolecules. We expect to gain insights into the spectra-structure correlation for a well-defined molecular structure characteristic for crystal lattices. To evaluate the impact of the model complexity on the accuracy of the simulated spectra, we consider a number of molecular models (monomers, dimers, multimers). This procedure allows for indirect investigation of the effects of chemical surrounding on NIR spectra. Obtained results are used to estimate of the vibrational contributions to NIR spectra of purines and pyrimidines. On this basis, we can discuss the differences between contributions of particular modes to NIR spectra. Finally, we intend to obtain insight into the relationships between intermolecular interactions of nucleobases and their NIR features. We expect that our results will increase the chemical specificity of NIR spectroscopy and its potential in examining various biological materials (e.g. plants, natural products)^30,31^, tissue examination in the context of biomedical diagnosis (e.g. cancer detection)^39,71,72^, and rapidly developing NIR hyperspectral imaging of biological samples^36–38^.

## Methods

### Experimental

Polycrystalline (powder) samples of nucleobases were purchased from Sigma-Aldrich (purity level: > 99% - adenine, cytosine, and thymine; > 98% - guanine) and milled before spectral measurements. Diffuse Reflectance (DRIFT) NIR spectra were measured on a Bruker Vector 22/N FT-NIR spectrometer. A spectrum of each sample was measured in the 10000–4000 cm^−1^ region, with spectral resolution of 4 cm^−1^ resulting in 2 cm^−1^ of interpolated data spacing. For each sample, the spectral measurement was carried out three times, and 128 scans were accumulated. The spectra were measured at 298 K and then converted to absorbance scale. No spectral pretreatment was necessary.

### Computational details

Multi-modal anharmonic computations were necessary in order to obtain data on the first overtones and binary combinations, which are the most influential contributions into NIR spectra. Due to an extensive computational cost of anharmonic approximation, it was required to represent the major structural features of crystalline nucleobases by simplified molecular models. We undertook a systematic approach - for each nucleobase we selected a number of structures of increasing complexity, from monomers to clusters consisting of up to six molecules. The initial molecular geometries of the clusters were extracted from the crystal structures available from Cambridge Structural Database (CSD)^73^. Further details of this approach will be shown in Results and Discussion Section.

Computations of NIR spectra were performed using Deperturbed Vibrational Second-Order Perturbation Theory (DVPT2) approach^43,44^. The determination of the ground-state electronic properties was conducted with Density Functional Theory (DFT) calculations at M06-2X level. M06-2X single-hybrid density functional is parametrized towards long-range interactions^74^, which were additionally refined by applying Grimme’s third version of empirical correction for dispersion (GD3)^75^. The following basis sets were applied: 6-311 ++ G(2df, 2pd) for monomers, and 6-31 G(d, p) for dimers and larger clusters. Additionally, these calculations were also done with the use of B3LYP functional. The comparison between M06-2X and B3LYP approaches revealed that the latter strongly overestimates the inter-layer interactions between nucleobase molecules in the clusters. This leads to flattening of the two-layered models into single-layer structures. On the other hand, ground state structures obtained with M06-2X functional relatively well reflects the crystalline state. Therefore, only the results of  M06-2X calculations are discussed in this work. All quantum mechanical calculations were performed with Gaussian 09 Rev. E01 software^76^.

The spectral lineshapes were modelled by Lorentz-Gauss (Cauchy-Gauss) product function^77,78^. The processing and visualization of the experimental and calculated data was done using script written in MATLAB (The MathWorks Co.).

## Results and Discussion

### Structural simulation of NIR spectra of solid state nucleobases

**Figure 1:**
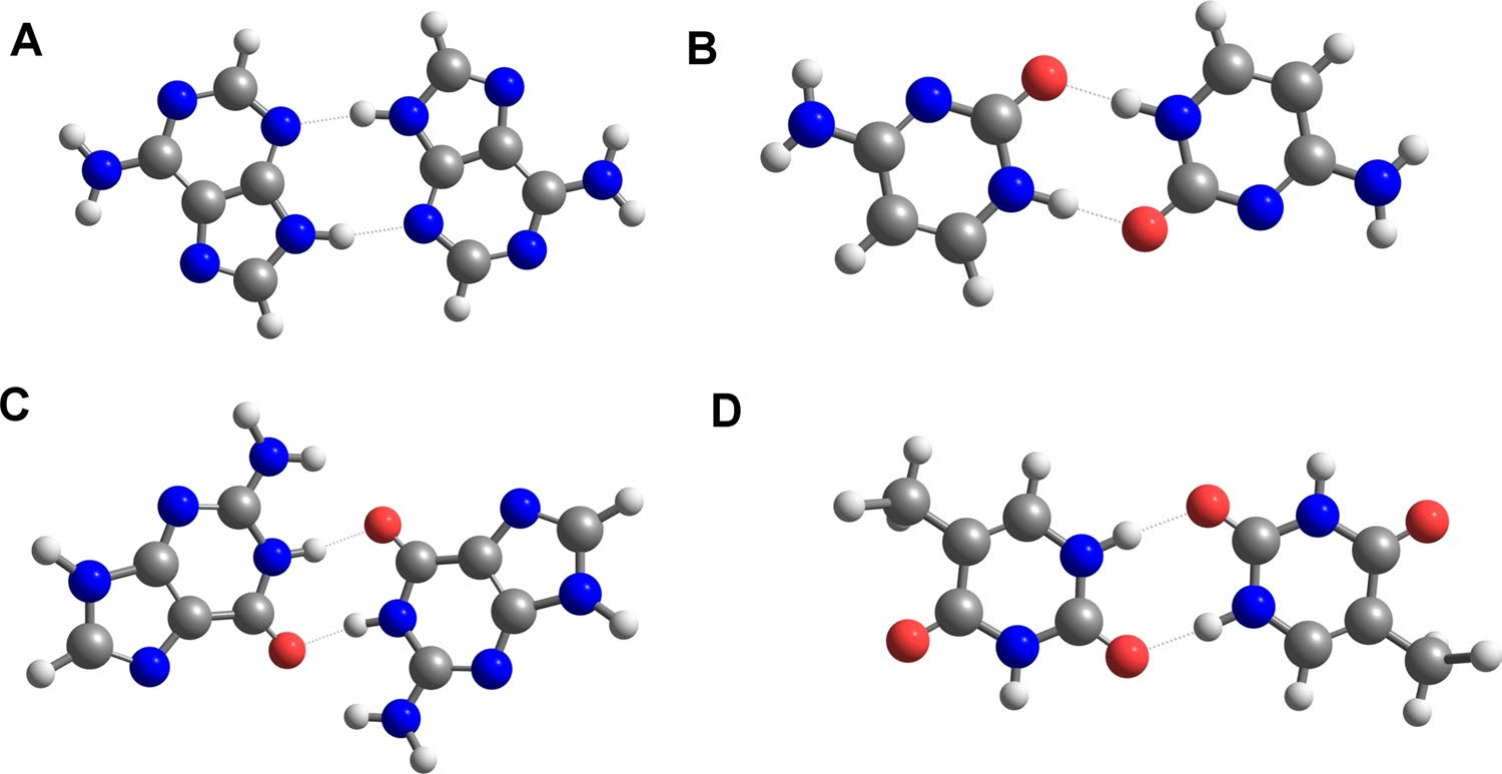
Dimer structures of (**A**) adenine; (**B**) cytosine; (**C**) guanine; (**D**) thymine.

**Figure 2:**
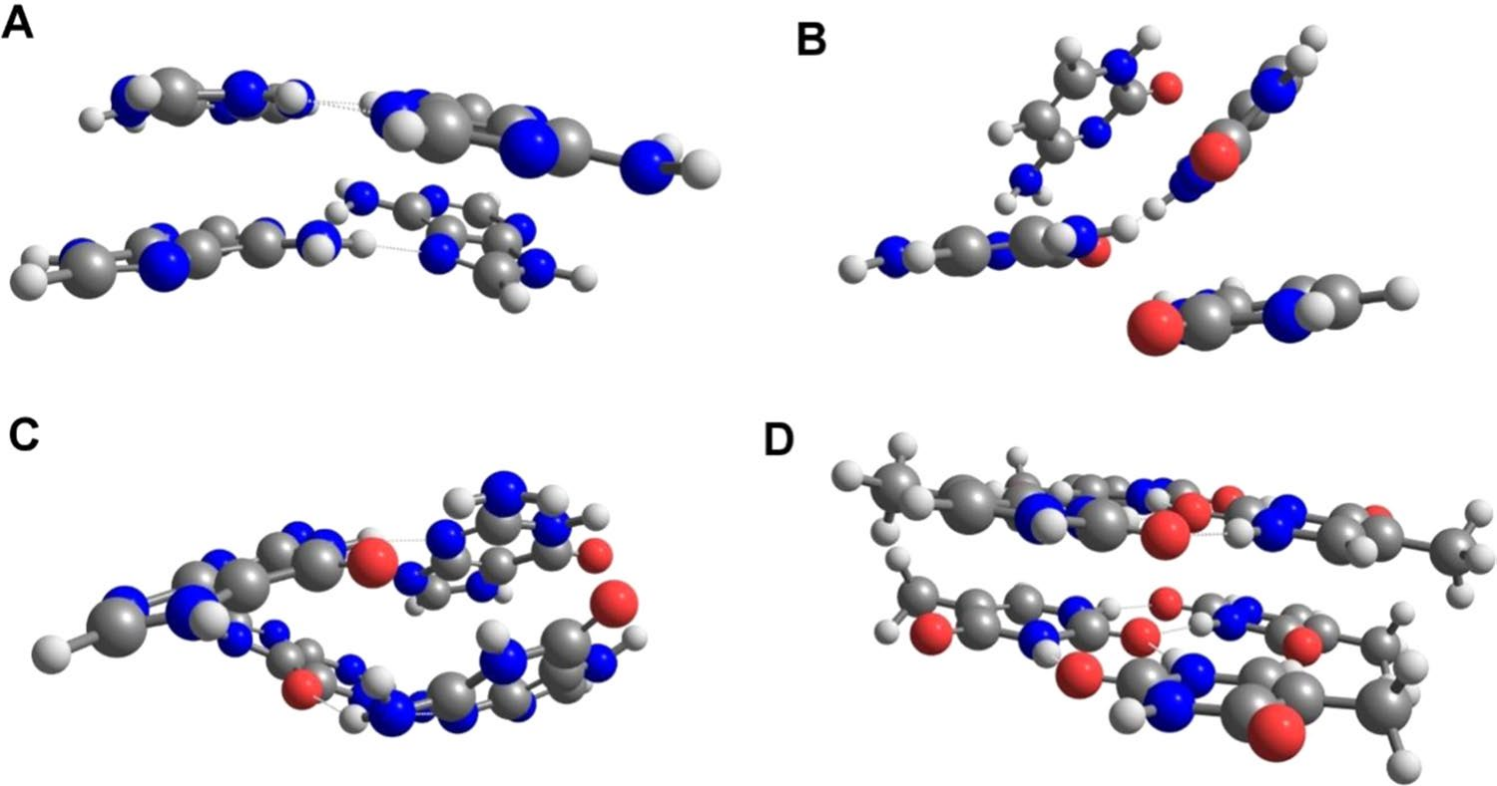
Molecular clusters of (**A**) adenine; (**B**) cytosine; (**C**) guanine; (**D**) thymine.

Chemical environment in a crystalline lattice is highly specific and different from that of non-interacting molecules diluted in solvents^23^ or well-defined complexes formed in solutions^53^. On the other hand, our previous investigation of polycrystalline melamine suggested that the simplification of molecular models does not reduce remarkably the accuracy of calculated overtones and combinations^50^. To some extent, the structure of melamine resembles that of the nucleobases. However, it is necessary to examine whether this similarity also appears in NIR and mid-IR vibrational features^50^. To verify this assumption, at first we calculated NIR spectra using single molecule models of adenine, guanine, cytosine, and thymine. This approximation completely neglects the chemical surrounding effect. Intermolecular interactions of nucleobases may roughly be separated into in-plane and inter-plane ones^79^. The former ones are stronger and predominantly controlled by hydrogen-bonding between the proton donor and acceptor centers of nucleobases^8^. The latter ones include ring stacking forces, which are relatively weaker^80^. We used the dimers as the simplest model that mimic in-plane (or *XY* plane) interactions (Fig. 1). We selected the most stable dimeric structures known in the literature^81^. To gain insight into the role of both kinds of interactions, at least four molecules in two layers are required (Fig. 2). The layered models include inter-layer (or *Z* plane) interactions as well.

Thus, we constructed four-molecules clusters by extracting their geometry from the crystalline structures. Prior to the vibrational analysis, it was mandatory to optimize these structures to reach the minimum at the potential energy surface. This step resulted in a modest distortion of the clusters in comparison with the crystalline structures. To minimize this effect, and keep the computational cost at an acceptable level, we have selected M06-2X-GD3/6-31 G(d, p) method that is computationally efficient and reasonably well describes the inter-molecular forces existing between the molecules of nucleobases. The obtained clusters adequately reproduced the major structural motifs existing in crystal lattices (Fig. 2). Molecules of pyrimidines are smaller and allow for calculation of considerably larger clusters. Thymine cluster consisting of six molecules was still acceptable for the anharmonic treatment. Unlike the case of cytosine, the larger cluster of thymine was only slightly distorted during the optimization. For this reason, we selected the six-molecule cluster of thymine for the present study (Fig. 2).

**Figure 3:**
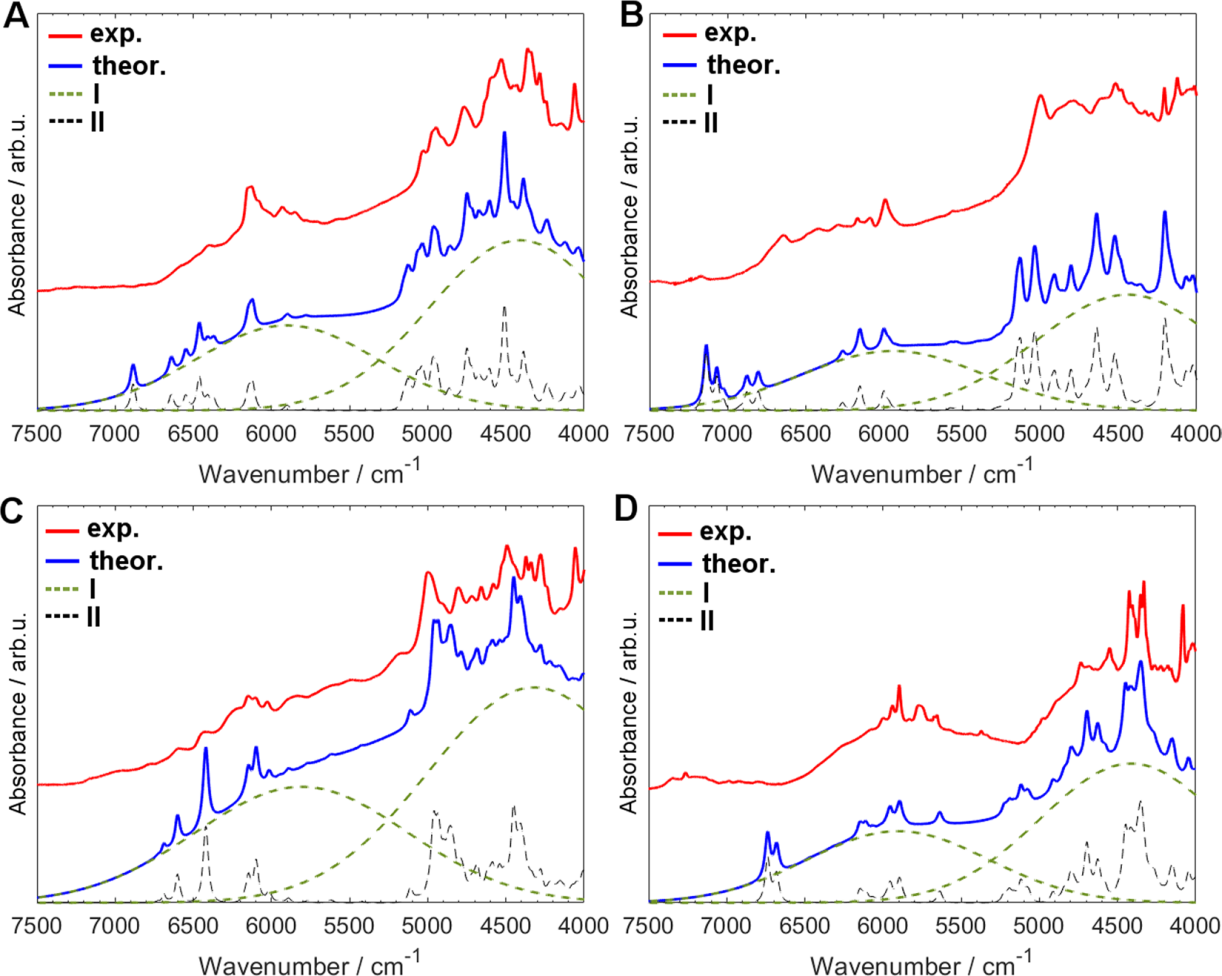
Experimental and simulated (DVPT2//M062X/6–311++G(2df, 2pd) NIR spectra of crystalline (**A**) adenine; (**B**) cytosine; (**C**) guanine; (**D**) thymine in the 4000–7500 cm^−1^ region. Approximated effects of the baseline contributions (I - green dashed lines) which are added to the theoretical spectrum (II - black dashed lines).

We considered a number of factors to improve the agreement between the simulated and experimental NIR spectra of crystalline nucleobases. Even simple inspection of the experimental spectra reveals a pronounced baseline elevation (Fig. 3A–D). This effect is observed in a broad spectral region (from 6700 to 4000 cm^−1^) for all four compounds. Similar effect was observed in NIR spectra of carboxylic acids^49,53–55^. These studies also evidenced the concentration dependence of the baseline fluctuation^49^. As in the case of carboxylic acids, one can notice an existence of at least two components contributing to the observed baseline fluctuation. This effect is the most pronounced in the spectrum of thymine (Fig. 3D) and adenine (Fig. 3A), while in the spectrum of cytosine (Fig. 3B) and guanine (Fig. 3C) the baseline is more uniformly elevated throughout entire NIR region. It is probable that the appearance of both components is related to strong hydrogen-bonding interactions present in the samples. However, the phenomenon responsible for the background elevation was not reproduced in our calculations. This is the main origin of the disagreement between the calculated and experimental values of band positions and intensities. At present, we are unable to propose an exhaustive explanation of this effect. It is interesting to note that such broad spectral features appear in NIR spectra of compounds that form cyclic dimers with strong hydrogen bonding^49,53–55^. One may speculate that the delocalization of the electron density is responsible for this broad absorption. However, additional studies are necessary to confirm this hypothesis. To improve the agreement between the experimental and theoretical spectra, the baseline was numerically fitted. For this purpose, we applied the same band model as that used for modeling of NIR bands^53^. Details of this procedure are presented in Supporting Information.

As expected, distinct differences in the theoretical NIR spectra appear when using either the monomers, dimer, or clusters for the calculations. Obviously, the models of monomers do not include intermolecular interactions, and thus are expected to be insufficient. Surprisingly, the models of clusters develop a number of additional bands, which are not present in the experimental spectra. The theoretical spectra combining monomers and dimers provided the best approximation of the experimental spectra. This conclusion is in line with the former simulations of NIR spectra of short-^53^ and medium-chain^54^ fatty acids. Here, by mixing the theoretical spectra of the dimer and monomer with 1:1 ratio we obtained a reasonably accurate reproduction of NIR spectra of crystalline nucleobases. To improve the analysis of the spectra, we applied uniform wavenumber scaling^82^ for each of the calculated spectra (adenine: 0.9811; cytosine: 0.9854; guanine: 0.9615; thymine: 0.9807). The spectra adjusted this way were used for further discussions. Note that the empirical frequency scaling factors are intended to correct all kinds of errors resulting from approximations that are necessarily applied in quantum chemical calculations. The purpose of the scaling factors is to adjust the calculated frequencies to match the experimental ones^83^. In this study, the application of scaling factors followed an observation that the meaningful calculated peaks have systematically overestimated positions. Hence, scaling was applied for better presentation of the spectra and to easy the discussion. A few factors may contribute to the observed overestimation of the calculated peak positions. Primarily, the inaccuracies in the potential energy resulting from approximations in the electronic theory used in our calculations, density functional theory and basis set combination. Moreover, this effect may also result from properties of VPT2 theory. Its computational efficiency is obtained through a relatively shallow probing of the vibrational potential. VPT2 approach predicts the shape of the potential based on its local curvature. This may result in an overestimation of X-H frequencies, e.g. as reported by us previously^56,57^.

### Contribution of various vibrational modes to NIR spectra of nucleobases

The calculated NIR spectra include the first overtones and binary combinations^56^. Our previous studies have shown that the contributions from higher quanta transitions to NIR spectra in the 10,000–4000 cm^−1^ region (e.g. second overtones and ternary combinations) are smaller than 20%^56^. Thus, the majority of spectral information in this range is well-reflected by the first overtones and binary combinations. As mentioned before, NIR spectra are complex with magnitude of overlapping peaks. To easy the spectral analysis of nucleobases, we used projections of the relative contributions of pre-selected modes as a function of wavenumber.

**Figure 4:**
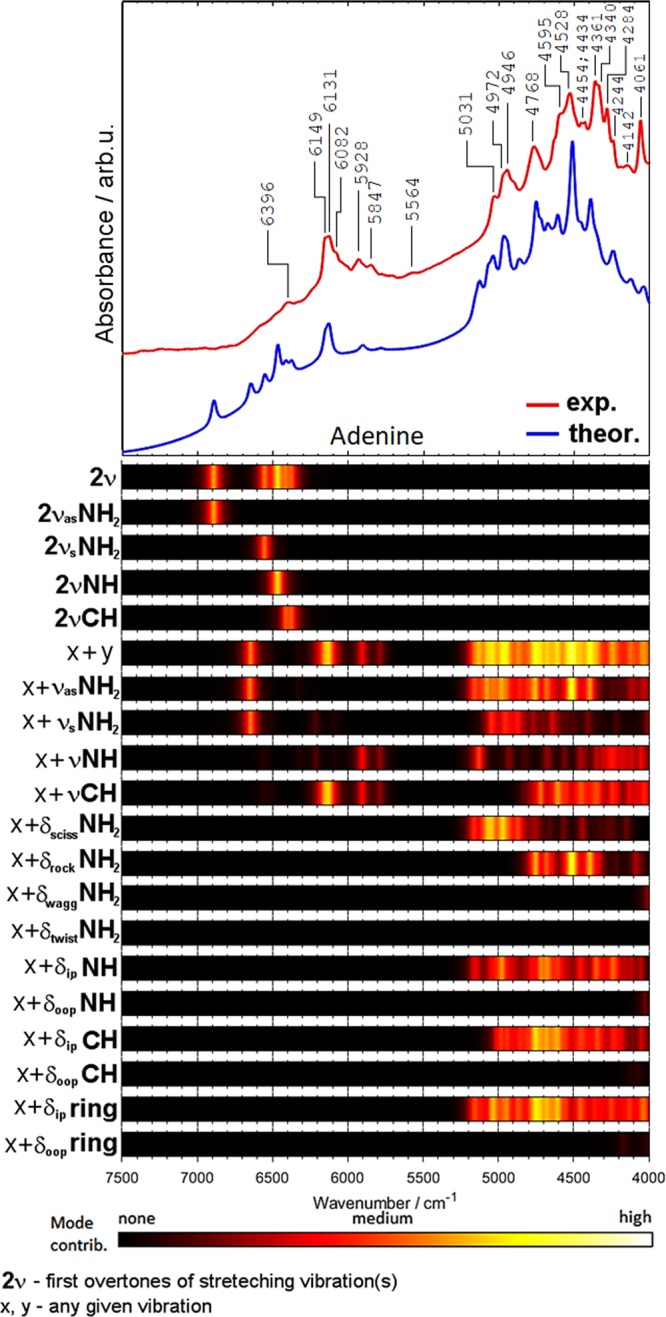
Comparison of the experimental and theoretical (DVPT2//M062X/6–311++G(2df, 2pd) NIR spectra of adenine together with the theoretical contributions of selected vibrational modes to NIR intensity.

**Figure 5:**
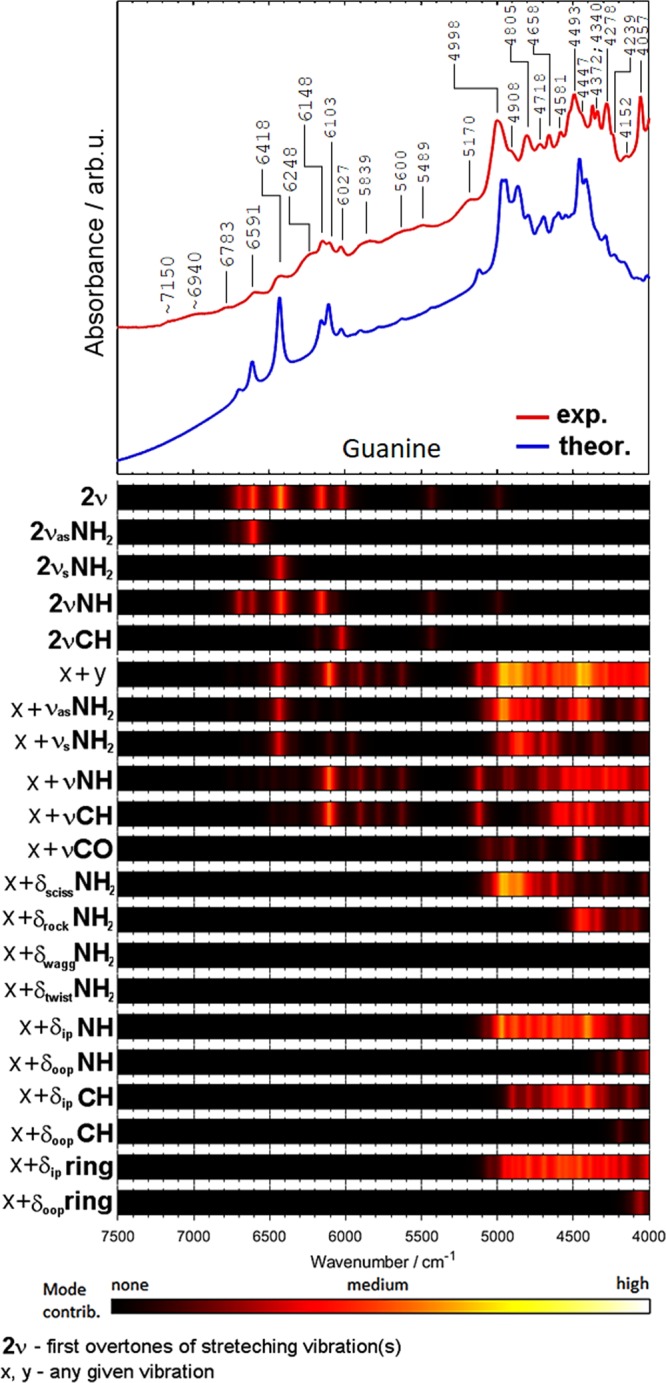
Comparison of the experimental and theoretical (DVPT2//M062X/6-311++G(2df, 2pd) NIR spectra of guanine together with the theoretical contributions of selected vibrational modes to NIR intensity.

**Figure 6:**
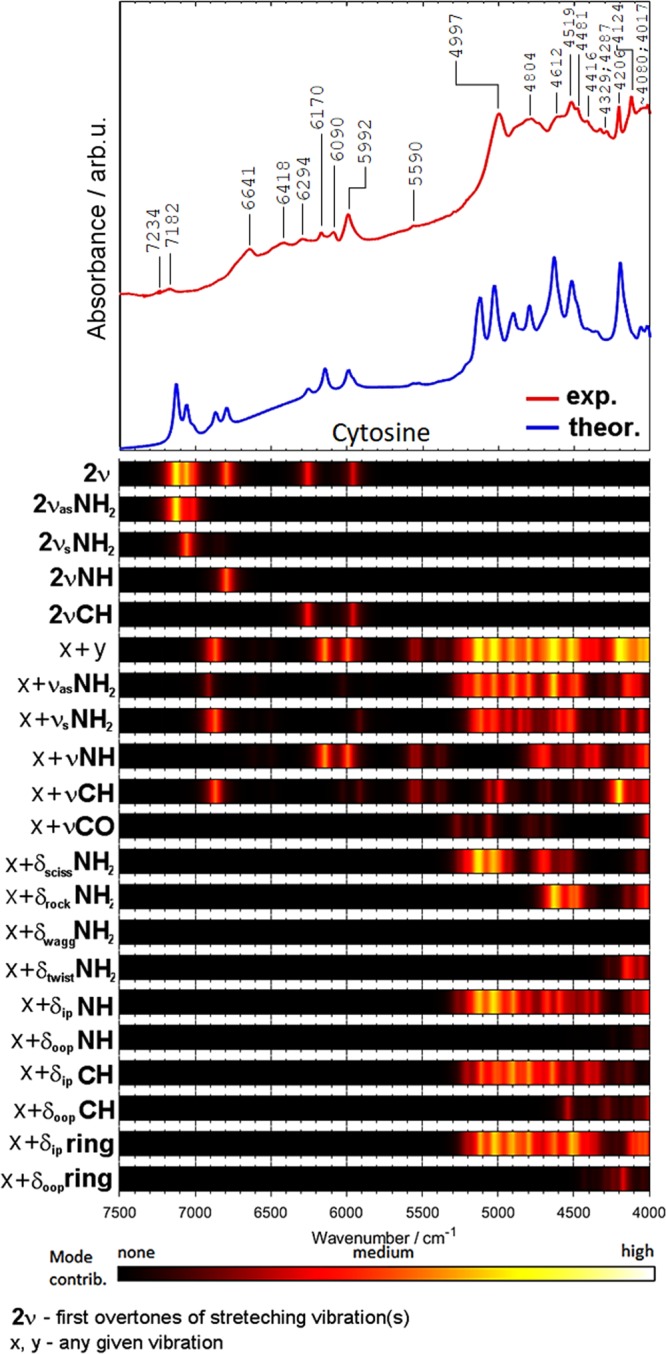
Comparison of the experimental and theoretical (DVPT2//M062X/6-311++G(2df, 2pd) NIR spectra of cytosine together with the theoretical contributions of selected vibrational modes to NIR intensity.

**Figure 7:**
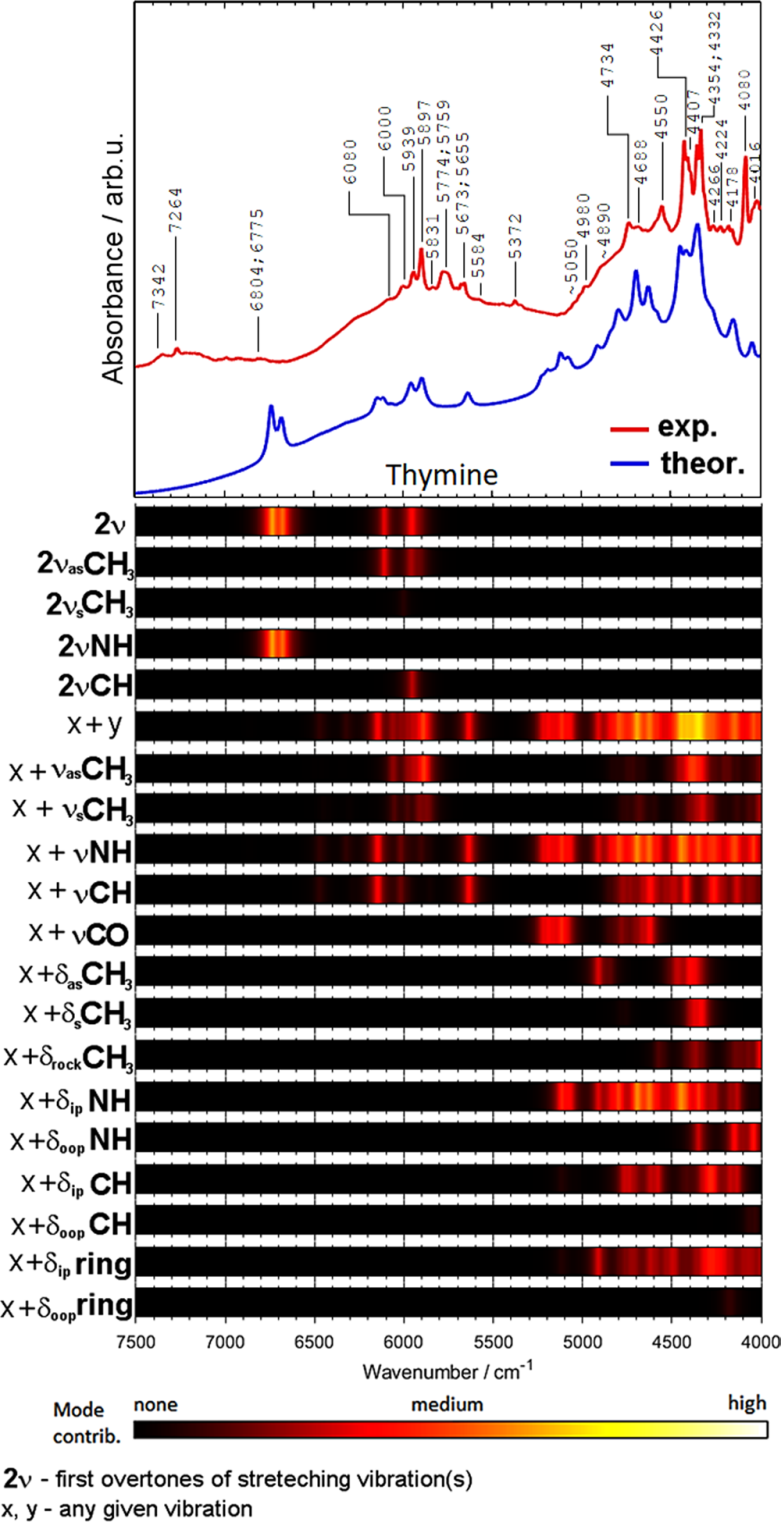
Comparison of the experimental and theoretical (DVPT2//M062X/6-311++G(2df, 2pd) NIR spectra of thymine together with the theoretical contributions of selected vibrational modes to NIR intensity.

Figures 4–7 present the contributions of selected vibrational modes to simulated NIR spectra of crystalline nucleobases. The first overtones (2*ν*) contribute predominantly in the 7500–5500 cm^−1^ region. As expected, the largest contributions should be assigned to the NH and NH_2_ stretching modes. In the region of 6500–5500 cm^−1^ one can also observe the combination bands resulting from stretching vibrations.

The region of 5800–4000 cm^−1^ is contributed almost entirely by the combination bands (*ν*_x_ + *ν*_y_; where *ν*_x_ and *ν*_y_ refer to any given stretching or bending mode). The most important are the combinations involving the *ν*NH and *ν*NH_2_ vibrations. The relative contributions are similar for all four nucleobases. As expected, the importance of the *ν*C=O combination bands is relatively low in NIR spectra, in contrast to mid-IR spectra of nucleobases, where these bands strongly absorb in the 1735–1667 cm^−1^ region^84–86^. Interestingly, thymine has two C=O moieties in its structure and the predicted *ν*C=O contribution to NIR spectrum (Fig. 7) is higher than that for cytosine (Fig. 6) and guanine (Fig. 5). In addition to the νC=O combination bands, further studies are necessary to explain a possible contribution from the second overtone of the C=O stretching vibration (3*ν*C=O). This band appears as a moderately intense feature near 5100 cm^−1^ in other molecules^33^.

The contributions of the combinations involving *δ*NH_2_ vibrations are significant for all four nucleobases. Rocking NH_2_ vibration is noticeable in the spectra of adenine and cytosine, but less evident in guanine. Bands from wagging and twisting NH_2_ vibrations do not appear in NIR spectra of nucleobases, except for cytosine, for which the contribution from the twisting mode is observed near 4000 cm^−1^ (Fig. 6).

The contributions from the NH deformation modes depend on the symmetry of vibration. In-plane vibrations are significant for all molecules, while out-of-plane ones are important only in the spectra of thymine (Fig. 7) and guanine (Fig. 5). The contributions from the in-plane CH deformation modes are more significant than those from the out-of-plane vibrations. It is expected since the in-plane deformation bands appear at higher wavenumbers than the out-of-plane ones. It is interesting to note that the contributions from the in-plane vibrations are stronger for nucleobases with purine rings (adenine, guanine) as compared with the nucleobases with the pyrimidine ring (cytosine, thymine). Similar trend one can observe for in-plane and out-of-plane ring vibrations. The in-plane vibrations are more important than the out-of-plane ones. In addition, the contributions from the in-plane vibrations are more significant for purines. These observations confirm a strong relationship between the molecular structure and the NIR spectrum.

Thymine is particularly intriguing because it features a CH_3_ group in its structure (Figs 1, 7). As can be seen, the contribution of the CH_3_ vibrations to NIR spectra remains moderate. The first overtones of the CH_3_ stretching modes contribute in relatively narrow wavenumber regions (Fig. 7). One can also notice that the combinations involving the *ν*CH_3_ and *δ*CH_3_ modes are rather weak (Fig. 7). Additionally, these bands are heavily overlapped with more pronounced combinations from the *ν*XH, *ν*C=O, and *δ*NH modes.

### NIR bands as vibrational markers of nucleobases

Systematic studies of mid-IR and Raman spectra resulted in establishing of the correlation tables and characteristic bands originating from purines and pyrimidines^86–89^. Therefore, nucleobases and their derivatives (e.g. corresponding nucleotides) can be discriminated in mid-IR and Raman spectra of biological samples. The bands appearing in the lower mid-IR region are particularly useful. For instance, Mello *et al*.^87^ have reported discrimination between the four nucleobase units in the spectra of DNA. The C=O stretching fundamental frequencies of guanine appear at 1710 and 1716 cm^−1^, while those of thymine are observed at 1700 and 1664 cm^−1^. Banyay *et al*.^88^ have reported a list of characteristic wavenumbers that provide highly specific information on nucleic acid structure, including pairing and stacking effects. Band shift and intensity changes in response to various effects, like conformational changes, intermolecular interactions or solvent effect are available in the literature^86,87,89^. Comparable libraries of Raman characteristic frequencies are accessible as well^90^. In contrast, similar data are not available for NIR region. Our calculations allow, for the first time, to establish the spectra-structure correlations in NIR region for nucleobases (Table 1).

**Table 1:** Characteristic NIR bands of nucleobases.

Characteristic bands (major contributions)	Nucleobase	Wavenumber (cm^−1^)
combinations involving *ν*NH	Thymine	4080
combinations involving *ν*CH	Cytosine	4206
combinations involving *ν*NH, *δ*_ip_NH, *ν*_as_CH_3_	Thymine	4354–4332
combinations involving *ν*CH, *δ*_rock_NH_2_, *δ*_ip_ring	Adenine	4361
combinations involving *ν*NH, *δ*_ip_NH	Thymine	4426
combinations involving *δ*_ip_NH, *δ*_ip_ring	Guanine	4493
combinations involving *ν*_as_NH_2_, *δ*_rock_NH_2_, *δ*_ip_ring	Cytosine	4519–4481
combinations involving *ν*_as_NH_2,_	Adenine	4528
combinations involving *ν*NH, *δ*_ip_NH, *δ*_rock_NH_2_	Thymine	4550
combinations involving *δ*_ip_CH, *δ*_ip_ring	Adenine	4768
combinations involving *ν*_as_NH_2,_ *δ*_sciss_NH_2_	Guanine	4908
combinations involving *δ*_sciss_NH_2,_ *δ*_ip_NH	Adenine	4972–4946
combinations involving *δ*_sciss_NH_2_, *δ*_ip_ring, *δ*_ip_NH	Cytosine	4997
combinations involving *δ*_ip_NH, *δ*_sciss_NH_2_	Guanine	4998
combinations involving *ν*_as_CH_3,_ *ν*_s_CH_3_	Thymine	5997
combinations involving *ν*NH	Cytosine	5992
combinations involving *ν*CH	Adenine	6149–6082
combinations involving *ν*NH, *ν*CH	Guanine	6148–6103
overtones of *ν*NH	Adenine	6670–6450
overtones of *ν*NH	Cytosine	6970–6720
overtones of *ν*NH, *ν*CH	Guanine	6940–6025
overtones of *ν*NH, *ν*CH	Thymine	6500–5890

We identify a number of specific combination bands of purines and pyrimidines (Table 1). This provides an opportunity to discriminate both structures in NIR spectra. From the point-of-view of chemical specificity, the overtone bands appear to be less useful. This results from the red-shift and broadening of the overtone bands due to hydrogen-bonding. The resulting broad, overlapping bands may become even less distinct in the spectra of biological samples where overlapping with the *ν*CH bands of lipids is anticipated^36–38^.

It seems that the combination bands involving the NH and NH_2_ vibrations are the most essential for identification of nucleobases. These bands have relatively high contributions to NIR spectra and are less likely to be obscured by the bands from other biomolecules, which makes them particularly useful in applications. The NH_2_ groups in purines (adenine, guanine) and cytosine develop sharp peaks in vicinity of 5030–4970 cm^−1^. NIR bands associated with the NH stretching and deformation vibrations are particularly specific for thymine. Besides, only thymine shows characteristic stretching bands due to the CH_3_ group (Table 1). These vibrations give rise to a distinct, sharp and intense doublet at 4426 and 4332 cm^−1^ (Fig. 7).

### General impact and possible applications of the present study

The present study demonstrates the feasibility of obtaining detailed insight into the origin of NIR bands of nucleobases through the quantum mechanical calculations. This approach helps to distinguish the bands of nucleic acids in NIR spectra of biological samples. Improvement of the chemical specificity and the capability of yielding structural insights is expected to enhance the potential of NIR spectroscopy in bioscience. For the first time, it was possible to establish a correlation table linking the frequencies of the characteristic NIR bands of nucleobases with their structural fragments.

Moreover, our results evidence that NIR bands are more sensitive to the effect of the nearest chemical environment than to the longer-range structural arrangement. This is in contrast to the trends observed for fundamental bands in mid-IR and Raman spectra^50^. Calculations based on relatively simple molecular models of nucleobases tend to reproduce accurately the experimental NIR bands in crystalline state. This observation is in contrast to simulated mid-IR spectra, where the molecular complexity needs to be reflected by the model much more precisely^50^. This creates an opportunity for efficient theoretical simulation of NIR spectra of other kinds of biomolecules.

The observed relatively lower sensitivity of NIR bands to the long-range interactions has a far-reaching impact. NIR spectra of nucleobases are sensitive to the local neighborhood of the molecule, but less sensitive to a more remote chemical environment. This observation suggests that NIR bands are better probes of e.g. nucleobase pairing, by being relatively less obscured by other effects when compared with mid-IR and Raman spectra. The hydrogen-bonding between nucleobases results in characteristic NIR spectral pattern including red-shift and substantial broadening of the overtone bands of NH and NH_2_ groups. This effects likely contribute to the profound baseline elevation in NIR spectra of nucleobases. Similar effect has also been observed in the spectra of fatty acids^53–55^. In contrast, combination bands, even those that involve *ν*NH/NH_2_ vibrations, are relatively less affected than the overtones.

## Conclusions

NIR spectra of adenine, guanine, cytosine, and thymine were reproduced by anharmonic calculations. The obtained results allow for detailed insight into the origin of NIR bands, the relationship with molecular structure and intermolecular interactions. The simulated first overtones and binary combination bands have been compared with the NIR spectra measured for polycrystalline samples. The calculations were based on a number of molecular models including monomers, dimers, and clusters consisting of four to six molecules. The most accurate approximation of the experimental spectra was accomplished by a combination of the theoretical spectra of the monomers and dimers with a subsequent wavenumber scaling.

Our results provide new insight into the origin of NIR bands of nucleobases. It appears that for all studied nucleobases the combinations bands are far more important than the overtone bands. The overtones of the NH stretching vibrations strongly contribute to the higher wavelength region of NIR spectra, while their binary combinations with the other modes influence considerably the region of 5200–4000 cm^−1^. The overtones and combination bands of low-lying fundamentals do not appear in NIR spectra of nucleobases, e.g. out-of-plane ring deformation modes. This is in agreement with the observation that the cluster models that include inter-plane interactions do not improve the accuracy of the simulated spectrum. Thus, the in-plane NIR modes of nucleobases are well approximated by model including the dimers only.

It is of note that more complex models of nucleobases provide poorer results as compared to smaller models. This result corresponds well with that obtained for melamine^50^. The exact description of crystalline state appears to be less important for obtaining of an accurate theoretical NIR spectrum than for the corresponding mid-IR spectrum^50^. The present study confirms that NIR bands are relatively less sensitive for the intermolecular interaction and chemical environment compared with the fundamental bands. This observation suggests that NIR spectroscopy may be used as an efficient tool for monitoring which substances cause direct mutations in living cells.

As yet, most of NIR spectroscopic studies of intermolecular interactions have been focused on overtones of X-H stretching vibrations. On the other hand, a very limited information is available on the other kinds of bands contributing to NIR spectra. We have demonstrated that neither combination bands nor overtones of deformation modes follow the pattern known for the overtones of stretching vibrations. Additionally, the profound baseline elevation effect was noticed. Similar effect was previously observed in NIR spectra of fatty acids. Two broad spectral features seem to be responsible for this effect. At present, the origin of these two components is not known and additional studies are required.

The correlation tables between NIR spectra and the structure of nucleobases obtained from the theoretical simulations is an important step towards expanding of potential of NIR spectroscopy in biomedical applications.

## Supplementary information

Supplemental information

## References

[1] Miescher F (1871). Ueber die chemische Zusammensetzung der Eiterzellen. Medicinisch-chemische Untersuchungen..

[2] Heard RD, Kinnersley HW, O’brien JR, Peters RA, Reader V (1933). Vitamin B_4_ and adenine. Nature.

[3] Xu W, Chan KM, Kool ET (2017). Fluorescent nucleobases as tools for studying DNA and RNA. Nat. Chem..

[4] Barth, A., Haris, P. I. Biological and biomedical infrared spectroscopy, Vol. 2 Advances in Biomedical Spectroscopy. *IOS Press BV*, *Amsterdam*, *Netherlands* (2009).

[5] Taillandier E, Liquier J (1992). Infrared spectroscopy of DNA. Methods Enzymol..

[6] Chalmers, J. M. & Griffiths, P. R. (Ed.). Handbook of vibrational spectroscopy. John Wiley & Sons, Chichester (2002).

[7] Stuart, B. H. Infrared spectroscopy of biological applications: an overview. John Wiley & Sons, New York (2011).

[8] Kyogoku Y, Lord RC, Rich A (1966). Hydrogen bonding specificity of nucleic acid purines and pyrimidines in solution. Science.

[9] Nir E, Kleinermanns E, de Vries MS (2000). Pairing of isolated nucleic-acid bases in the absence of the DNA backbone. Nature.

[10] Mons M, Dimicoli I, Piuzzi F, Tardivel B, Elhanine M (2002). Tautomerism of the DNA base guanine and its methylated derivatives as studied by gas-phase infrared and ultraviolet spectroscopy. J. Phys. Chem. A.

[11] Shimanouchi, T., Tsuboi, M. & Kyogoku, Y. Infrared spectra of nucleic acids and related compounds. In Advances in chemical physics: structure & properties of biomolecules, Volume 7, Duchesne, J. (Ed.) John Wiley & Sons, Inc, London, (1964).

[12] Madzharova F, Heiner Z, Gühlke M, Kneipp J (2016). Surface-enhanced hyper-Raman spectra of adenine, guanine, cytosine, thymine, and uracil. J.Phys.Chem. C.

[13] Gąsior-Głogowska M, Malek K, Zajac G, Barańska M (2016). A new insight into the interaction of cisplatin with DNA: ROA spectroscopic studies on the therapeutic effect of the drug. Analyst.

[14] Rome, C. *et al*. Near-infrared optical imaging of nucleic acid nanocarriers *in vivo*. In: Ogris, M., Sami, H. (Eds) *Nanotechnology for nucleic acid delivery*. *Methods in molecular biology*, vol 1943. Humana Press, New York, NY (2019). 10.1007/978-1-4939-9092-4_23 30838628

[15] Reva I, Nowak MJ, Lapinski L, Fausto R (2012). Spontaneous tunneling and near-infrared-induced interconversion between the amino-hydroxy conformers of cytosine. J. Chem. Phys..

[16] Lacher JR, CampIon DE, Park JD (1949). Near infrared absorption spectra of uracil, 5-chlorouracil, and thymine. Science.

[17] Gabelica, V. (Ed.) Nucleic acids in the gas phase, Springer-Verlag Berlin Heidelberg, (2016).

[18] Rein, R., Shibata, M., Garduno-Juarez, R. & Kieber-Emmons, T. Structure and dynamics: nucleic acids and proteins, Adenine Press, New York, pp. 269–288 (1983). 10.1080/07391102.1983.10507502 6400904

[19] Siesler, H. W., Ozaki, Y., Kawata, S. & Heise, H. M. (Eds), Near-infrared spectroscopy, Wiley-VCH: Weinheim (2002).

[20] Ozaki, Y., Huck, C. W. & Beć, K. B. Near-IR spectroscopy and its applications. In: *Molecular and laser spectroscopy*. *Advances and applications*. Gupta, V. P. (Ed.); San Diego, Calif.: Elsevier, p. 11–38 (2018).

[21] Cozzolino, D. (Ed.). Infrared spectroscopy: Theory, developments and applications. UK ed. Nova Science Pub Inc (2014).

[22] Czarnecki MA, Morisawa Y, Futami Y, Ozaki Y (2015). Advances in molecular structure and interaction studies using near-infrared spectroscopy. Chem. Rev..

[23] Grabska J, Beć KB, Ozaki Y, Huck CW (2017). Temperature drift of conformational equilibria of butyl alcohols studied by near-infrared spectroscopy and fully anharmonic DFT. J. Phys. Chem. A.

[24] Gonjo T, Futami Y, Morisawa Y, Wójcik MJ, Ozaki Y (2011). Hydrogen bonding effects on the wavenumbers and absorption intensities of the OH fundamental and the first, second and third overtones of phenol and 2,6-dihalogenated phenols studied by visible/near-infrared/infrared spectroscopy and density functional theory calculations. J. Phys. Chem. A.

[25] Chatani E, Tsuchisaka Y, Masuda Y, Tsenkova R (2014). Water Molecular System Dynamics Associated with Amyloidogenic Nucleation as Revealed by Real Time Near Infrared Spectroscopy and Aquaphotomics. Plos One.

[26] Huck, C. W. Infrared spectroscopy in near-infrared/infrared bioanalysis including imaging, John Wiley & Sons, Encyclopedia of Analytical Chemistry, (2016).

[27] Ozaki, Y. *et al*. Near-infrared spectroscopy in biological molecules and tissues. In: Roberts G., Watts A., European Biophysical Societies (Eds) Encyclopedia of biophysics. Springer, Berlin, Heidelberg (2019).

[28] Ciurczak, E. W. & Drennen, J. K. III Pharmaceutical and medical applications of near-infrared spectroscopy, CRC Press, Boca Raton (2002).

[29] Dorrepaal R, Gowen A (2018). Identification of magnesium oxychloride cement biomaterial heterogeneity using Raman chemical mapping and NIR hyperspectral chemical imaging. Sci. Rep..

[30] Türker-Kaya S, Huck CW (2017). A review of mid-infrared and near-infrared imaging: principles, concepts and applications in plant tissue analysis. Molecules.

[31] Cozzolino D (2009). Near infrared spectroscopy in natural products analysis. Planta Med..

[32] Huck CW (2015). Advances of infrared spectroscopy in natural product research. Phytochem. Lett..

[33] Chen Y (2014). Combined IR/NIR and density functional theory calculations analysis of the solvent effects on frequencies and intensities of the fundamental and overtones of the C=O stretching vibrations of acetone and 2-hexanone. J. Phys. Chem. A.

[34] Chan KLA, Kazarian SG (2016). Attenuated total reflection Fourier-transform infrared (ATR-FTIR) imaging of tissues and live cells. Chem. Soc. Rev..

[35] Ishigaki M, Yasui Y, Puangchit P, Kawasaki S, Ozaki Y (2016). *In vivo* monitoring of the growth of fertilized eggs of medaka fish (Oryzias latipes) by near-infrared spectroscopy and near-infrared imaging—a marked change in the relative content of weakly hydrogen-bonded water in egg yolk just before hatching. Molecules.

[36] Ishigaki M (2017). Noninvasive, highspeed, near infrared imaging of the biomolecular distribution and molecular mechanism of embryonic development in fertilized fish eggs. J. Biophotonics..

[37] Ishigaki M, Kawasaki S, Ishikawa D, Ozaki Y (2016). Near-infrared spectroscopy and imaging studies of fertilized fish eggs: *in vivo* monitoring of egg growth at the molecular level. Sci. Rep..

[38] Ishigaki M (2018). Nonstaining blood flow imaging using optical interference due to doppler shift and near-infrared imaging of molecular distribution in developing fish egg embryos. Anal. Chem..

[39] Ozaki, Y. *et al*. Near-infrared spectroscopy in biological molecules and tissues. In Roberts G., Watts A. (Eds), European biophysical societies. Encyclopedia of biophysics. Springer, Berlin, Heidelberg (2018).

[40] Ma L (2019). systematic discovery about NIR spectral assignment from chemical structural property to natural chemical compounds. Sci. Rep..

[41] Siesler H.W. (2017). Near-Infrared Spectra, Interpretation. Encyclopedia of Spectroscopy and Spectrometry.

[42] Beć, K. B. & Huck, C. W. Breakthrough potential in near-infrared spectroscopy: spectra simulation. A review of recent developments. *Front*. *Chem*. **7**, Article 48 (2019). 10.3389/fchem.2019.00048 PMC6396078 30854368

[43] Beć, K. B., Grabska, J. & Ozaki, Y. Advances in anharmonic methods and their applications to vibrational spectroscopies. In Frontiers of quantum chemistry; Wójcik, M. J., Nakatsuji, H., Kirtman, B., Ozaki, Y.; (Eds); Springer: Singapore, p.438–512 (2017).

[44] Beć, K. B., Grabska, J., Huck, C.W. & Ozaki, Y. Quantum mechanical simulation of near-infrared spectra. Applications in physical and analytical chemistry. In Molecular spectroscopy: a quantum chemistry approach. Ozaki, Y., Wójcik, M. J., Popp, J.; (Eds); Wiley-VCH, Weinheim, Germany; p. 353–388 (2019).

[45] Beć KB, Futami Y, Wójcik MJ, Ozaki Y (2016). A spectroscopic and theoretical study in the near-infrared region of low concentration aliphatic alcohols. Phys. Chem. Chem. Phys..

[46] Beć KB, Grabska J, Czarnecki MA (2018). Spectra-structure correlations in NIR region: spectroscopic and anharmonic DFT study of *n*-hexanol, cyclohexanol and phenol. Spectrochim. Acta A.

[47] Beć KB, Karczmit D, Kwaśniewicz M, Ozaki Y, Czarnecki MA (2019). Overtones of νCN vibration as a probe of structure of liquid CH3CN, CD_3_CN, and CCl_3_CN: combined infrared, near-infrared, and Raman spectroscopic studies with anharmonic density functional theory calculations. J. Phys. Chem. A.

[48] Barone V (2015). CC/DFT route toward accurate structures and spectroscopic features for observed and elusive conformers of flexible molecules: pyruvic acid as a case study. J. Chem. Theory Comput..

[49] Beć KB, Futami Y, Wójcik MJ, Nakajima T, Ozaki Y (2016). Spectroscopic and computational study of acetic acid and its cyclic dimer in the near-infrared region. J. Phys. Chem. A.

[50] Grabska J, Beć KB, Kirchler CG, Ozaki Y, Huck CW (2019). Distinct difference in sensitivity of NIR vs. IR bands of melamine to inter-molecular interactions with impact on analytical spectroscopy explained by anharmonic quantum mechanical study. Molecules.

[51] Beć KB, Grabska J, Kirchler CG, Huck CW (2018). NIR spectra simulation of thymol for better understanding of the spectra forming factors, phase and concentration effects and PLS regression features. J. Mol. Liq..

[52] Kirchler CG (2017). Critical evaluation of spectral information of benchtop vs. portable near-infrared spectrometers: Quantum chemistry and two-dimensional correlation spectroscopy for a better understanding of PLS regression models of the rosmarinic acid content in Rosmarini folium. Analyst.

[53] Grabska J, Ishigaki M, Beć KB, Wójcik MJ, Ozaki Y (2017). Structure and near-infrared spectra of saturated and unsaturated carboxylic acids. An insight from anharmonic DFT calculations. J. Phys. Chem. A.

[54] Grabska J, Beć KB, Ishigaki M, Wójcik MJ, Ozaki Y (2017). Spectra-structure correlations of saturated and unsaturated medium-chain fatty acids. Near-infrared and anharmonic DFT study of hexanoic acid and sorbic acid. Spectrochim. Acta A.

[55] Grabska J, Beć KB, Ishigaki M, Huck CW, Ozaki Y (2018). NIR spectra simulations by anharmonic DFT- saturated and unsaturated long-chain fatty acids. J. Phys. Chem. B.

[56] Grabska J, Czarnecki MA, Beć KB, Ozaki Y (2017). Spectroscopic and quantum mechanical calculation study of the effect of isotopic substitution on NIR spectra of methanol. J. Phys. Chem. A.

[57] Beć KB, Grabska J, Huck CW, Czarnecki MA (2019). Spectra–structure correlations in isotopomers of ethanol (CX_3_CX_2_OX; X = H, D): combined near-infrared and anharmonic computational study. Molecules.

[58] Bloino J, Baiardi A, Biczysko M (2016). Aiming at an accurate prediction of vibrational and electronic spectra for medium-to-large molecules: An overview. Int. J. Quantum Chem..

[59] Biczysko M, Panek P, Barone V (2009). Toward spectroscopic studies of biologically relevant systems: Vibrationalspectrum of adenine as a test case for performances of long-range/dispersioncorrected density functionals. Chem. Phys. Lett..

[60] Puzzarini C, Biczysko M, Barone V (2011). Accurate anharmonic vibrational frequencies for uracil: the performance of composite schemes and hybrid CC/DFT model. J. Chem. Theory Comput..

[61] Fornaro T, Biczysko M, Montia S, Barone V (2014). Dispersion corrected DFT approaches for anharmonic vibrational frequency calculations: nucleobases and their dimers. Phys. Chem. Chem. Phys..

[62] Puzzarini C (2013). Accurate molecular structure and spectroscopic properties of nucleobases: a combined computational–microwave investigation of 2-thiouracil as a case study. Phys. Chem. Chem. Phys..

[63] Fornaro T, Burini D, Biczysko M, Barone V (2015). Hydrogen-bonding effects on infrared spectra from anharmonic computations: uracil–water complexes and uracil dimers. J. Phys. Chem. A.

[64] Krasnoshchekov SV, Vogt N, Stepanov NF (2015). Ab Initio anharmonic analysis of vibrational spectra of uracil using the numerical-analytic implementation of operator Van Vleck perturbation theory. J. Phys. Chem. A.

[65] Thomas PS, Carrington T, Agarwal J, Schaefer HF (2018). Using an iterative eigensolver and intertwined rank reduction to compute vibrational spectra of molecules with more than a dozen atoms: Uracil and naphthalene. J. Chem. Phys..

[66] Minaeva V, Karaush-Karmazin N, Baryshnikov G, Minaev B (2019). A complete characterization of vibrational IR and Raman spectra of the highly-symmetrical octathia[8]circulene. Vibrational Spectroscopy.

[67] Minaeva VA (2015). Temperature effects in low-frequency Raman spectra of corticosteroid hormones. Opt. Spectrosc..

[68] Minaeva VA, Minaev BF, Baryshnikov GV, Romeyko OM, Pittelkow M (2013). The FTIR spectra of substituted tetraoxa[8]circulenes and their assignments based on DFT calculations. Vibrational Spectroscopy.

[69] Michal P (2019). Vibrational optical activity of intermolecular, overtone, and combination bands: 2-chloropropionitrile and α-pinene. J. Phys. Chem. B.

[70] Pecul, M. & Sadlej, J. Chiral Recognition by Molecular Spectroscopy. In *Molecular spectroscopy: a quantum chemistry approach*. Ozaki, Y., Wójcik, M. J., Popp, J.; (Eds.); Wiley-VCH, Weinheim, Germany; p. 171–196 (2019).

[71] Huck CW, Huck-Pezzei VAC, Ozaki Y (2016). Critical review upon the role and potential of fluorescence and near-infrared imaging and absorption spectroscopy in cancer related cells, serum, saliva, urine and tissue analysis. Curt. Med. Chem..

[72] Huck CW (2016). Highly efficient novel vibrational spectroscopic methods – food, medicinal plant, material and cancer analysis. GIT – Laboratory Journal.

[73] Groom CR, Bruno IJ, Lightfoot MP, Ward SC (2016). The Cambridge Structural. Database. Acta Cryst..

[74] Zhao Y, Truhlar DG (2008). The M06 suite of density functionals for main group thermochemistry, thermochemical kinetics, noncovalent interactions, excited states, and transition elements: two new functionals and systematic testing of four M06-class functionals and 12 other functionals. Theor. Chem. Acc..

[75] Grimme S, Antony J, Ehrlich S, Krieg H (2010). A consistent and accurate ab initio parameterization of density functional dispersion correction (DFT-D) for the 94 elements H-Pu. J. Chem. Phys..

[76] Gaussian 09, Revision E.01, Frisch, M. J. *et al*. Gaussian, Inc., Wallingford CT, (2013).

[77] Grabska J, Ishigaki M, Beć KB, Wójcik MJ, Ozaki Y (2017). Correlations between structure and near-infrared spectra of saturated and unsaturated carboxylic acids. Insight from anharmonic density functional theory calculations. J. Phys. Chem. A.

[78] Bradley MS (2015). Lineshapes in IR and Raman spectroscopy: a primer. Spectroscopy.

[79] Bravaya KB, Kostko O, Ahmed M, Krylov AI (2010). The effect of π-stacking, H-bonding, and electrostatic interactions on the ionization energies of nucleic acid bases: adenine–adenine, thymine–thymine and adenine–thymine dimers. Phys. Chem. Chem. Phys..

[80] Sigel A, Operschall BF, Sigel H (2014). Comparison of the π-stacking properties of purine versus pyrimidine residues. Some generalizations regarding selectivity. J. Biol. Inorg. Chem..

[81] Schlathçlter T (2006). Ion-induced biomolecular radiation damage: from isolated nucleobases to nucleobase clusters. Chem. Phys. Chem..

[82] Scott AP, Radom L (1996). Harmonic vibrational frequencies:  an evaluation of Hartree–Fock, Møller–Plesset, quadratic configuration interaction, density functional theory, and semiempirical scale factors. J. Phys. Chem..

[83] Johnson RD, Irikura KK, Kacker RN, Kessel R (2010). Scaling factors and uncertainties for ab initio anharmonic vibrational frequencies. J. Chem. Theory Comput..

[84] Mathlouthi M, Seuvre AM (1984). F.T.-I.R. and laser-Raman spectra of thymine and thymidine. Carbohydrate Research.

[85] Mathlouthi M, Seuvre AM (1986). F.T.-I.R. and laser-Raman spectra of guanine and guanosine. Carbohydrate Research.

[86] Mathlouthi M, Seuvre AM, Koenig JL (1986). F.T.-I.R. and laser-Raman spectra of cytosine and cytidine. Carbohydrate Research.

[87] Mello MLS, Vidal BC (2012). Changes in the infrared microspectroscopic characteristics of DNA caused by cationic elements, different base richness and single-stranded form. PLoS One.

[88] Banyay M, Sarkarb M, Graslund. A (2003). A library of IR bands of nucleic acids in solution. Biophysical Chemistry.

[89] Movasaghi Z, Rehman S (2008). ur Rehman, I. Fourier transform infrared (FTIR) spectroscopy of biological tissues. Appl. Spectrosc. Rev..

[90] Olsztyska-Janus Sylwia, Gsior-Gogowska Marlena, Szymborska-Maek Katarzyna, Czarnik-Matusewicz Bogusawa, Komorowsk Magorzata (2011). Specific Applications of Vibrational Spectroscopy in Biomedical Engineering. Biomedical Engineering, Trends, Research and Technologies.

