# Author Correction: Simulated NIR spectra as sensitive markers of the structure and interactions in nucleobases

**DOI:** 10.1038/s41598-020-57956-1

**Published:** 2020-01-16

**Authors:** Krzysztof B. Beć, Justyna Grabska, Yukihiro Ozaki, Mirosław A. Czarnecki, Christan W. Huck

**Affiliations:** 1 Institute of Analytical Chemistry and Radiochemistry, Leopold-Franzens University, Innrain 80/82, CCB-Center for Chemistry and Biomedicine, 6020 Innsbruck, Austria; 2 Department of Chemistry, School of Science and Technology, Kwansei Gakuin University, Sanda, Hyogo, 669-1337 Japan; 3 Faculty of Chemistry, University of Wrocław, F. Joliot-Curie 14, 50-383 Wrocław, Poland

Correction to: *Scientific Reports* 10.1038/s41598-019-53827-6, published online 22 November 2019

This Article contains typographical errors in the Acknowledgements section.

“This work was partially supported by the National Science Center Poland, Grant 2016/21/B/ST4/02102”.

should read:

“This work was partially supported by the National Science Center Poland, Grant 2017/27/B/ST4/00948”

