# Supplementary material for: Simulated NIR spectra as sensitive markers of the structure and interactions in nucleobases
Source: Sci Rep. 2019 Nov 22;9:17398. doi: 10.1038/s41598-019-53827-6 (PMC6874539; doi:10.1038/s41598-019-53827-6)
Supplement: Supplementary file 1 — Supplemental information [file 41598_2019_53827_MOESM1_ESM.pdf]

Supplementary Information

Simulated NIR spectra as sensitive markers of the structure and interactions in nucleobases

Krzysztof B. Beć,[1]* Justyna Grabska,[1] Yukihiro Ozaki,[2] Mirosław A. Czarnecki,[3] Christan W. Huck[1]

[1] *Institute of Analytical Chemistry and Radiochemistry, Leopold-Franzens University, Innrain 80/82, CCB-Center for Chemistry and Biomedicine, 6020 Innsbruck, AUSTRIA*

[2] *Department of Chemistry, School of Science and Technology, Kwansei Gakuin University, Sanda, Hyogo 669-1337, JAPAN*

[3] *Faculty of Chemistry, University of Wrocław, F. Joliot-Curie 14, 50-383 Wrocław, POLAND*

* Corresponding author: K.B. Beć, email address: krzysztof.bec@uibk.ac.at

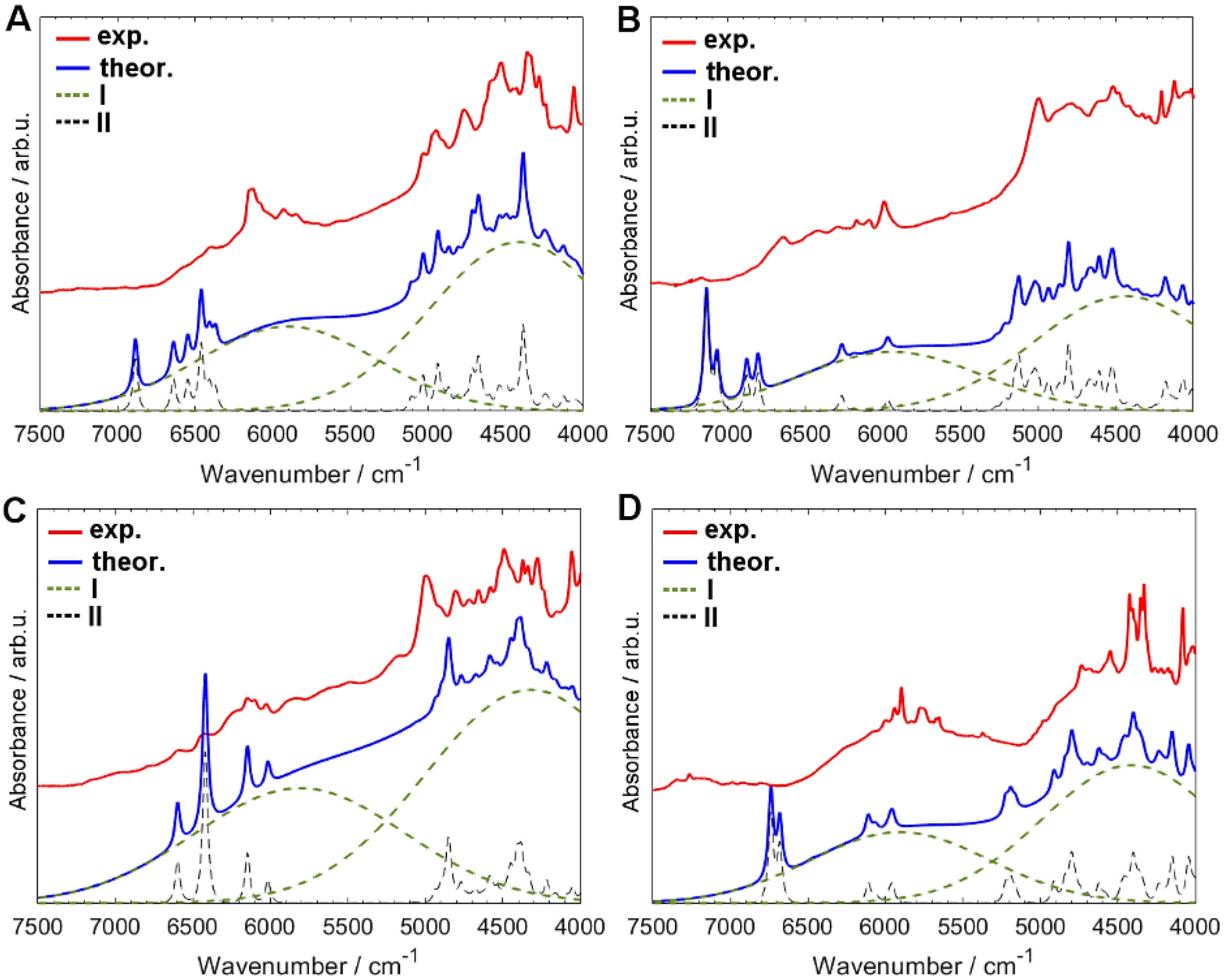

Figure S1. Experimental and simulated NIR spectra of nucleobases in 4000-7500 cm^{-1} region. Simulated spectra are based entirely on monomer models. (A) adenine; (B) cytosine; (C); guanine; (D) thymine, in crystalline phase. Approximated effect of the baseline contributions (I - green dashed lines) which is added to the theorotical spectrum (II - black dashed lines).

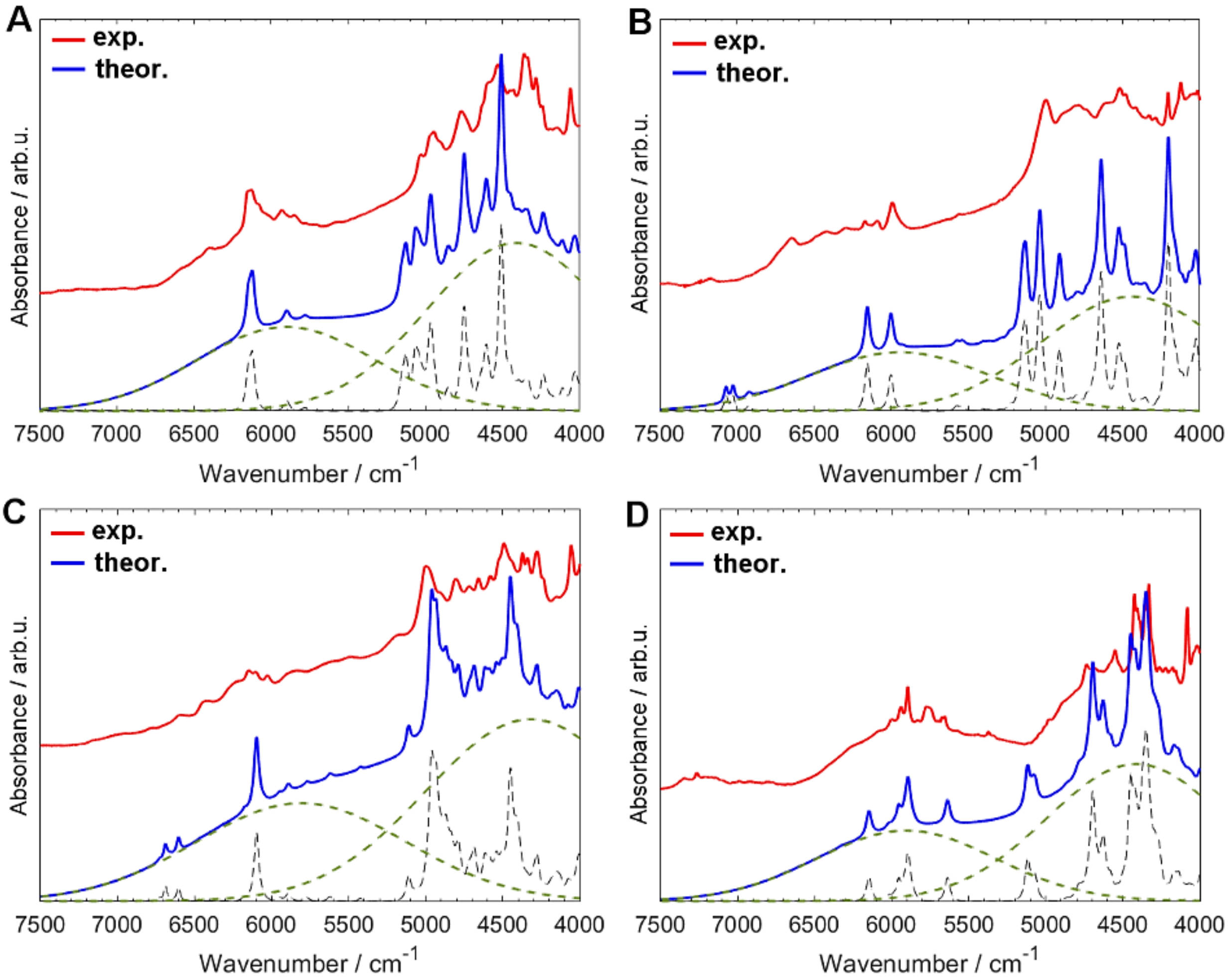

Figure S2. Experimental and simulated NIR spectra of nucleobases in 4000-7500 cm^{-1} region. Simulated spectra are based entirely on dimer models. (A) adenine; (B) cytosine; (C); guanine; (D) thymine, in crystalline phase. Approximated effect of the baseline contributions (I - green dashed lines) which is added to the theorotical spectrum (II - black dashed lines).

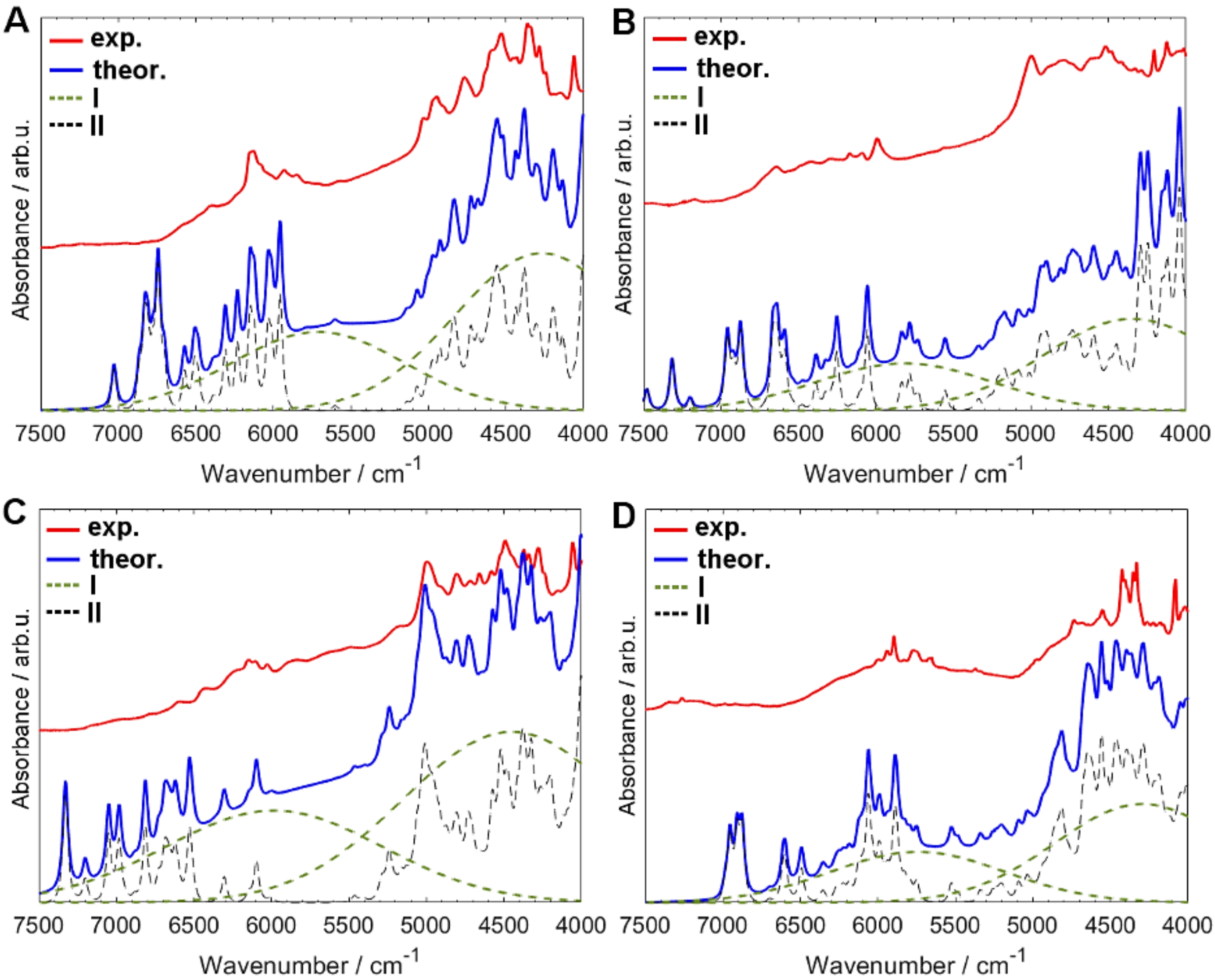

Figure S3. Experimental and simulated NIR spectra of nucleobases in 4000-7500 cm^{-1} region. Simulated spectra are based entirely on cluster models. (A) adenine; (B) cytosine; (C); guanine; (D) thymine, in crystalline phase. Approximated effect of the baseline contributions (I - green dashed lines) which is added to the theorotical spectrum (II - black dashed lines).

Appendix I. Details of the baseline fitting procedure

The baseline elevation effect observed in NIR spectra of nucleobases was reproduced by using similar approach as the one used for broadening of the simulated bands (as explained in the main article).

It was carried out in the following procedure:

1. Cauchy-Gauss product function as the spectral profile was used in this work.
2. Starting parameters a_1-a_4 were chosen arbitrarily in such way, that approximate positions of the baseline maxima and elevation level roughly matched those observed in the experimental spectra.
3. The two-component baseline was added to the simulated spectrum.
4. A numerical optimization algorithm was run to fit the model spectrum to the experimental spectrum.
5. The fitting procedure was done in a straightforward way, by least-squares minimization of the difference between the experimental spectrum and the model spectrum.
6. For each of the processed spectra, the objective function had 8 parameters, the minimization was performed with the use of Powell gradientless algorithm. We have adopted this procedure from our previous work:
   Hawranek, J.P.; Grabska, J.; Beć, K.B. On optimizaion of absorption – dispersion spectra. *J. Mol. Struct*. **2016**, 1126, 11-18.
7. The optimized/fitted shape parameters are presented beneath (Table S2).

The above procedure was carried out using our code developed in MATLAB. We should note here, that the results of the band fitting carried out here should be treated in qualitative way, as the main aim was to reproduce the general feature of the experimental spectra, in the form of baseline elevation.

Table S2. Parameters of Cauchy-Gauss product funtion resutlign from band fitting/optimization procedure perforfmed to approximate the baseline elevation (details in the note above).

nucleobase	optimized/fitted parameters			
	a_1	a_2	a_3	a_4
adenine	6025	3.105	1.002E-05	0.0013
	4501	6.004	1.004E-05	0.0017
cytosine	6021	1.802	1.007E-05	0.0012
	4505	3.514	1.004E-05	0.0014
guanine	6052	3.504	1.017E-05	0.0010
	4509	6.511	1.080E-05	0.0011
thymine	6022	1.789	1.016E-05	0.0012
	4507	3.533	1.001E-05	0.0010

<u>Appendix II.</u> Details of the computational procedure for the presentation of the vibrational contributions in the simulated NIR spectra

Numerous overlapping bands populate NIR spectra of even relatively simple molecules (e.g. Refs. 42-43 in the main article). This makes their detailed analysis difficult, and the presentation of the band assignments is problematic. It is often impossible to distinguish a clearly leading contribution for a given NIR band.

To present the band assignments in a more informative way, and to easy the analysis of NIR spectra we propose the following method. We developed a density map of spectral contributions to better elucidate these influences and to present them in a clear manner.

The color in these maps corresponds to the square rooted intensity ratio of the selected simulated bands to the total intensity of modelled spectrum at any given wavenumber νi and additionally in proportion to the calculated intensity at that point.

The following formula is used for the determination of the relative contributions:

$$\text{contribution of j-th mode at } \nu_i = \sqrt[2]{\frac{\text{intensity of j-th simulated band at } \nu_i}{\text{sum of intensities of all simulated bands at } \nu_i}}$$

The square root was introduced to visually promote lesser contributions, otherwise they tend to be difficult to spot in the scale of the figure. The calculated value range from 0 (no contribution) to 1 (the NIR spectrum is influenced by the selected mode/modes entirely) for every wavenumber in the spectrum. These values are mapped to a selected colormap as in the legend bar presented underneath the figure. Hence, the color may be directly interpreted as the intensity of a given mode at a given wavenumber.

Acknowledgement

This work was supported by the Austrian Science Fund (FWF), M2729-N28.

This work was partially supported by the National Science Center Poland, Grant 2016/21/B/ST4/02102.

Calculations have been carried out in Wroclaw Centre for Networking and Supercomputing (http:/www.wcss.pl), under Grant no. 163.
